# Age at menarche, eating disorders, and their relationships with some parameters in female adolescents in Iran

**DOI:** 10.1186/s13104-021-05482-2

**Published:** 2021-02-25

**Authors:** Lida Daeie-Farshbaf, Mehrangiz Ebrahimi-Mameghani, Parvin Sarbakhsh, Neda Roshanravan, Ali Tarighat-Esfanjani

**Affiliations:** 1 Student Research Committee, School of Nutrition and Food Sciences, Tabriz University of Medical Sciences, Tabriz, Iran; 2 Nutrition Research Center, School of Nutrition and Food Sciences, Tabriz University of Medical Sciences, Attar Nishabouri St., POBOX: 14711, Tabriz, 5166614711 Iran; 3 Statistic and Epidemiology Research Center, Tabriz University Medical Sciences, Tabriz, Iran; 4 Cardiovascular Research Center, Tabriz University of Medical Sciences, Tabriz, Iran

**Keywords:** Adolescent, Anthropometry, Eating disorders, Girls, Menarche

## Abstract

**Objective:**

Adolescence as one of the most challenging periods of humans’ growth is accompanied with major physical, behavioural, social-emotional, and neuroendocrine changes. Early maturation and eating disorders (EDs) have been reported to be associated with adverse health conditions. Therefore, the present study was conducted to assess age of onset of menarche (AM), EDs, and their possible relationships with weight, body mass index (BMI), waist circumference (WC), and socioeconomic status (SES) in the Iranian female adolescents.

**Results:**

In the study population, mean ± standard deviation (SD) of AM was 12.95 ± 1.14 years. Inverse significant relationships were found between weight and AM, also BMI and AM (*p* < 0.05). There was a negative association between weight and anorexia nervosa (AN), BMI and AN, also WC and AN (*p* < 0.001). A significant positive correlation was found between SES and AM, also EDs and AM (p < 0.05) then between weight and bulimia nervosa (BN) & binge-eating disorder (BED), BMI and BN & BED, also WC and BD & BED (*p* < 0.001). Our results also revealed that AM in mothers had a significant positive relationship with AM in their daughters (*p* < 0.001, r = 0.34).

## Introduction

Menarche is often considered as a hallmark of puberty among females [1]. The age of menarche (AM) is of great importance in terms of clinical, public health, and social factors [2]. In the last two decades, a downward trend has been reported in the median age at menarche (14 years) in less-developed countries as well as some well-developed countries [3]. Early maturation has been found to be associated with adverse health conditions and development of a broad range of psychopathological symptoms like eating disorders (EDs) during adolescence [4–6]. Overall, early maturation has greater effects for girls in comparison with boys as reported by a study that has demonstrated the elevated internalizing and externalizing symptoms during early and mid-adolescence along with the elevated risk for EDs in mid and late adolescence [7]. Also, early menarche could accelerate skeletal maturation leading to short height of the adults [8].

Strong evidence shows that the increased rate of obesity in children is a significant factor contributing to early onset of menarche [9]. Matkovic et al. [10] showed that gain in body fat of 1 kg results in a 13-day reduction in AM. The adolescents with EDs frequently have menstrual abnormalities reflecting their abnormal nutritional intake [11]. Amenorrhea occurs in 68% of the individuals associated with weight loss, rigorous sports-related activities, and extreme calorie restriction [12, 13]. Although, several studies have found an association between age of pubertal development and body mass index (BMI) [14–16], others have failed to find any association in this regard [17, 18]. Studying menstrual disorders is an opportunity for early diagnosis and treatment of the people with EDs [11].

According to review of the literature, there was different and somewhat contradictory evidence regarding AM and EDs and their relationship with anthropometric parameters, and socioeconomic status (SES) so, herein, it was attempted to study AM and EDs and their relationship with weight, BMI, waist circumference (WC), and SES in the Iranian female adolescents.

## Main text

### Methods

This cross-sectional study included female students aged 14–18 years that were selected randomly from public and private high schools in all five educational districts during academic year of 2015–2016 in Tabriz, Iran (n = 725). Students with any endocrine disorders, hormonal disorders, or delayed menarche were excluded from the study.

Purpose of the study and instructions for completing questionnaires were explained for the selected students in a training session. An informed written consent was obtained from all the participants and their guardian.

#### Data collection

All the participants were asked to complete a researcher-made questionnaire containing demographic and SES characteristics. SES was assessed by collecting data regarding parents’ educational and occupational situation, number of family members, and type of school, and was categorized into “low”, “moderate”, and “high” subgroups. An Eating Disorders Diagnostic Scale (EDDS) was used based on diagnostic and statistical manual of mental disorders, fourth edition (DSM-IV) for diagnosis of EDs [19]. Validity of this questionnaire has been previously tested by Khabir [20]. This questionnaire contains a set of 23 self-report items assessing three categories of disorders including anorexia nervosa (AN), bulimia nervosa (BN), and binge-eating disorder (BED). Computer coding was used for scoring the questionnaire. Age of the participants was calculated based on the date of birth. Height, and weight were measured according to the standardized protocols [21] using the calibrated instruments (Stadiometer SECA model 213, SECA Corp., Hamburg, Germany, 2008) and (Beurer scale, model GS 202, Beurer GmbH., Germany, 2015), respectively. BMI was calculated as weight (kg) divided by the square of height (m^2^). WC; the distance around the narrowest area of the waist between the lowest rib and iliac crest at the end of a gentle expiration; was measured using a measuring tape. The age- and sex-specific BMI cut-off points were used, provided by the Centre for Disease Control and Prevention (CDC) according to which the BMI between 85th and 95th percentiles was classified as overweight and BMI above than 95th percentile was considered as obese [22]. AM was recorded for all the participants and their mothers.

#### Statistical analysis

Results were expressed as mean ± standard deviation (SD) for continuous variables and frequencies (%) for categorical ones. Data normality was checked using Kolmogorov–Smirnov test. The association between AM and anthropometric parameters was assessed using linear regression. Analyses of Variance (ANOVA) and Tukey’s post hoc were used to compare the mean ± SD of anthropometric parameters between AM groups as well as ED’s subgroups. The distribution of socioeconomic characteristics in EDs subgroups was determined by the chi-square test. SPSS software (The Statistical Package for the Social Science, version 23.0, IBM Corp., US, 2016) was used for all statistical analyses, and *p* value < 0.05 was considered as statistically significant.

### Results

Mean age was 15.83 ± 0.99 years and 75.7% of the students were 15 years old. Mean of AM in girls was 12.95 ± 1.14 years (n = 724). After the classification of participants into three groups based on the AM: (1) 12 < AM ≤ 14, (2) 14 < AM ≤ 16 and (3) 9 ≤ AM ≤ 12, the mean of weight, WC, and BMI in group one were significantly higher than group three (Table 1), however there was no difference in the mean of height between three groups. To recognize the factors associated with AM, linear regression analysis was performed (Table 2). Results showed that there was an inverse significant relationship between weight, BMI, and AM. Menstruation seemed to occur earlier in girls with higher weight or BMI (*p* < 0.05, 131 r = 0.1). This trend was shown in Additional file 2: Fig. S1A. The data indicated that AM was not significantly related to height and WC. Although, AM had a statistically significant positive correlation with SES, as with increment in the level of SES, AM increased (*p* < 0.05) (Additional file 2: Fig. S1B). We found that there was a statically significant relationship between the AM for girls and their mothers, as 1-month increase in maternal AM caused 4 months delaying of girl’s AM (r = 0.34, *p* < 0.001). Distribution of EDs among all participants was as follow: AN: 11.7% (n = 85), BN: 5.2% (n = 38), and BED: 0.7% (n = 5). Because of the low prevalence of BED, we supposed BED and BN subgroups as one group. Table 3 illustrates the mean values and SD of AM and anthropometric measurements in control and EDs groups. Analysis showed positive and inverse significant relationships of weight, BMI, WC with BN & BEDs and AN subgroups respectively. No significant relationship was seen between height and EDs groups. A significant correlation was found between AM and EDs as AM was occurred later in EDs subgroups.

**Table 1:** Mean (SD) of basic characteristics in girls based on groups of AM (n = 724^a^)

Variables	1 9 ≤ AM ≤ 12 n = 232	2 12 < AM ≤ 14 n = 437	3 14 < AM ≤ 16 n = 55	Total
Height (cm)	160.40 (5.83)	160.81 (5.58)	161.02 (4.94)	160.69 (5.61)
Weight (kg)	60.03 (10.72)^b^	57.21 (10.66)^b^	56.66 (13.39)	58.06 (10.98)
Waist circumference (cm)	74.12 (8.27)^c^	72.51 (7.87)^c^	73.08 (8.43)	73.07 (8.06)
Body mass index (kg/m^2^)	23.32 (3.97)^b, d^	22.09 (3.83)^d^	21.84 (4.75)^b^	22.46 (3.99)
Mothers’ AM (years)	13 (1.47)^d^	13.65 (1.07)^c, d^	14.36 (0.99)^c, d^	13.50 (1.27)

Groups are based on the age at which menstruation was occurred

*AM* age at menarche

^a^One person was ignored because of delayed menarche

^b^p < 0.01

^c^p < 0.05

^d^p < 0.001

**Table 2:** Regression analysis of AM and basic characteristics of girls (n = 724)

Variables	p value	β (95% CI)
Weight (kg)	< 0.01	− 0.01 (− 0.019, − 0.004)
Height (cm)	0.2	0.01 (− 0.005, 0.025)
WC (cm)	0.1	− 0.009 (− 0.019, 0.002)
BMI (kg/m^2^)	< 0.001	− 0.03 (− 0.06, − 0.018)
Mother's AM (year)	< 0.001	0.3 (− 0.0242, 0.381)
SES	< 0.05	0.1 (0.003, 0.322)

*AM* age at menarche, *WC* waist circumference, *BMI* body mass index; SES: socioeconomic status, *CI* confidence interval

Linear regression was performed; p < 0.05

**Table 3:** Mean (SD) of AM and anthropometric measurements in control and eating disorder groups

Variables	CONT (n = 597)	ED (n = 128)	
		AN (n = 85)	BN & BED (n = 38)
AM (years)	12.91 (1.1)	13.20 (1.07)	13.04 (1.1)*
BMI (kg/m^2^)	23.02 (3.6)	17.48 (1.5)	24.64 (4.5)**
Weight (kg)	59.48 (10.1)	45.11 (5.3)	64.20 (12.2)**
Height (cm)	160.68 (5.6)	160.48 (5.03)	161.34 (5.7)***
WC (cm)	73.95 (7.6)	65.20 (4.7)	76.50 (10.1)**

*AM* age at menarche, *AN* anorexia nervosa, *BN & BED* bulimia nervosa & binge-eating disorder, *BMI* body mass index, *WC* waist circumference

Analysis of Variance (ANOVA) was performed; p < 0.05

^*^p < 0.05

^**^p < 0.001

^***^Not significant

Distribution of socioeconomic characteristics in EDs subgroups has been shown in Additional file 1: Table S1. As shown in the Table, there was a significant relationship between normal and EDs groups as regards father’s income category (*p* = 0.03). Most of the participants’ fathers had middle income. The relationship between normal and EDs groups with regards mother’s education was also significant (*p* = 0.01). Most of the participants’ mothers belonged to “Diploma” and “Elementary and illiterate” subgroups in normal and EDs groups, respectively. SES in the most of the participants was determined as “middle” and with regards to this parameter, the relationship between normal and EDs groups was close to significant (*p* = 0.05).

### Discussion

In the present study, mean ± SD of AM was 12.95 ± 1.14 and 13.5 ± 1.27 154 years for girls and their mothers, respectively. Ghergherechi and Shoari in a study conducted in Tabriz (2011) showed that mean ± SD of AM was 12.58 ± 1.30 and 13.22 ± 1.22 years for girls and their mothers, respectively [23]. Results of both studies indicated that mean AM of girls has been decreased compared to that of mothers over the years. Seemingly, this decrease can be explained by changes in lifestyles and increase in overweight and obesity rate between the two generations.

In the present study, AM was inversely correlated with weight and BMI, which was similar to the study by Tehrani et al. [24], who found that overweight girls experienced menarche earlier than obese ones. Several studies [16, 18, 23, 25–27] have also shown that BMI was inversely correlated with AM but Bazrafshan et al. [17], found no significant association neither between BMI and AM nor weight and AM.

Our results demonstrated no significant associations between height and AM, which was in line with other studies [3, 16]. Unlike our results, Okasha et al. [28], and Farahmand et al. [18], reported that height had a positive significant correlation with AM. Our results also did not find any relationship between WC and AM but mean WC was higher in group one (9 ≤ AM ≤ 12) compared to the others. However, other studies have shown inverse association between WC and AM [16, 18]. These discrepancies could be due to demographic and genetic differences between study populations.

Our results showed a significant positive correlation between AM and SES, which was in contrast to the studies by Mee-Hwa Lee et al. [3], Gharavi et al. [29],and Delvarianzadeh et al. [25], who did not find any significant relationship between SES and AM. Asgharnia et al. [26], showed that subjects in upper socioeconomic class experienced menarche earlier. Krieger et al. [2], indicated that although mean AM is declining but patterns vary with respect to socioeconomic strata. Since, there are no standard tools for measuring SES; researchers mainly rely on the tools designed by them, which may lead to different results in studies. AM of the mothers is one of the strongest predictors of AM in their daughters. Herein, a positive strong correlation was observed between daughters’ and maternal AM, which was in accordance with the findings reported by other studies [23, 24, 30], suggesting that AM between two generations is partly controlled by genetic pattern [30].

Our results demonstrated significant relationships between weight, BMI, WC, and EDs subgroups so that, weight, BMI, and WC were lower in AN group and higher in BN and BED groups. These associations have been also found in earlier studies [31, 32]. In the AN group, having an energy-restricted diet and trying to lose weight were found to result in being underweight and excessive consumption of food and the lack of control on the amount of the consumed food in the BN and BED groups were found to result in being overweight or obese.

No significant relationship was observed between height and EDs subgroups. According to review of the literature, there was no study investigated the effects of EDs on height of the people. Since, this study was a cross-sectional study and height changes are time-dependent, thus prospective studies are needed to understand whether EDs can lead to short stature. Results of the current study revealed that AM occurred later in EDs subgroups, which was in contrast to the studies by Yannakoulia et al. [31], and Almuhlafi et al. [33], who observed that subjects with early menarche had higher scores of ED behaviours. This can be explained by the fact that a decrease in body fat mass may cause menstrual abnormalities and it is believed that discrepancies in results may be due to geographical and demographic differences of the subjects.

The findings of this study indicated an inverse association between father’s income, mother’s education, and SES with EDs as EDs were found to be lower in 197 subjects with higher father’s income, mother’s education and SES strata. Yannakoulia et al. [31], did not find any association between socio-demographic characteristics and eating attitudes. As mentioned earlier, it can be due to different definitions of SES in the studies.

### Conclusion

Due to the reduction of AM and its relationship with unfavourable psychopathological health conditions like EDs, determining factors affecting AM, control related risk factors and reproductive health education should be considered as important health system priorities.

## Limitations

The role of pre-existing psychiatric disorders or family history of a psychiatric disorder was not considered in this study, and it was not possible to determine its effects on the results, thus it was regarded as the main limitation of this study.

## Supplementary Information

**Additional file 1: Table S1.** Distribution of socioeconomic characteristics in Eating Disorders (EDs) subgroups (n = 725).

**Additional file 2: Figure S1.** (A) Age at menarche (AM) depending on body mass index (BMI). AM had an inverse significant relationship with BMI, as menstruation was occurred earlier in girls with higher BMI. (B) Age at menarche (AM) depending on socio-economic status (SES). AM had a statistically significant positive correlation with SES, as with increment in the level of SES, AM increased.

## Data Availability

The dataset analyzed during the current study is available from the corresponding author on reasonable request.

## Abbreviations

AM: Age onset of menarche
AN: Anorexia nervosa
ANOVA: Analyses of Variance
BED: Binge eating disorders
BMI: Body mass index
BN: Bulimia nervosa
CDC: Center for Disease Control and Prevention
EDDS: Eating Disorder Diagnostic Scale
EDs: Eating disorders
SES: Socioeconomic status
WC: Waist circumference
