# Supplementary material for: Age at menarche, eating disorders, and their relationships with some parameters in female adolescents in Iran
Source: BMC Res Notes. 2021 Feb 25;14:72. doi: 10.1186/s13104-021-05482-2 (PMC7905913; doi:10.1186/s13104-021-05482-2)
Supplement: Supplementary file 1 — Additional file 1: Table S1. Distribution of socioeconomic characteristics in Eating Disorders (EDs) subgroups (n = 725). [file 13104_2021_5482_MOESM1_ESM.docx]

| **Table S1- Distribution of socioeconomic characteristics in Eating Disorders (EDs) subgroups (n=725)** | | | | |
| --- | --- | --- | --- | --- |
| ***Characteristic*** | **Normal** | **EDs (n=128)** | |  |
|  | n=597 **(**82.3%) | *^a^*AN  n=85 (11.7% ) | *^b^* BN & BED  n=38 (5.2%) & n=5 (0.7%) | *P* |
| ***Father’s education***  University degree  Diploma  Elementary and illiterate | 90 (15.1)  236 (44.2)  242 (40.7) | 13 (15.3)  30 (35.3)  42 (49.4) | 7 (16.3)  24 (55.8)  12 (27.9) | ***^c^* Ns** |
| ***Father’s income category***  very low  Low  Middle  High  Very high | 12 (2)  176(29.6)  318 (53.4)  77 (12.9)  12 (2) | 2 (2.4)  18 (21.2)  46 (54.1)  13 (15.3)  6 (7.1) | 0  5 (11.6)  30 (69.8)  7 (16.3)  1 (2.3) | **0.03** |
| ***Mother’s education***  University degree  Diploma  Elementary and illiterate | 56 (9.4)  239 (49.2)  246 (41.3) | 6(7.1)  32(37.6)  47 (55.3) | 3 (7)  20 (46.5)  20 (46.5) | **0.01** |
| ***Mother’s occupation***  Housewives  Retired  Employee  Self-employed | 534 (89.9)  5 (0.8)  25 (4.2)  30 (5.1) | 80 (94.1)  1 (1.2)  3 (3.5)  1 (1.2) | 38 (88.4)  1 (2.3)  3 (7)  1 (2.3) | **Ns** |
| ***Household size***  ***<4***  ***≥4*** | 395 (66.4)  200 (33.6) | 57 (67.1)  28 (32.9) | 32 (74.4)  11 (88.4) | **Ns** |
| ***SES***  Low  Middle  High | 101 (17)  427 (71.8)  67 (11.3) | 7 (8.2)  73 (85.9)  5 (5.9) | 6 (14)  34 (79.1)  3 (7) | **0.05** |
| *^a^AN: Anorexia Nervosa, ^b^BN: Bolimia Nervosa, BED: Binge Eating Disorder/  ^c^Ns: Not significance*  *Significance level was considered p< 0.05/ Chi- square test was used* | | | | |
