# Supplementary material for: Age at menarche, eating disorders, and their relationships with some parameters in female adolescents in Iran
Source: BMC Res Notes. 2021 Feb 25;14:72. doi: 10.1186/s13104-021-05482-2 (PMC7905913; doi:10.1186/s13104-021-05482-2)
Supplement: Supplementary file 2 — Additional file 2: Figure S1. (A) Age at menarche (AM) depending on body mass index (BMI). AM had an inverse significant relationship with BMI, as menstruation was occurred earlier in girls with higher BMI. (B) Age at menarche (AM) depending on socio-economic status (SES). AM had a statistically significant positive correlation with SES, as with increment in the level of SES, AM increased. [file 13104_2021_5482_MOESM2_ESM.docx]

**Additional files**

**Additional file 2: Figure S1**


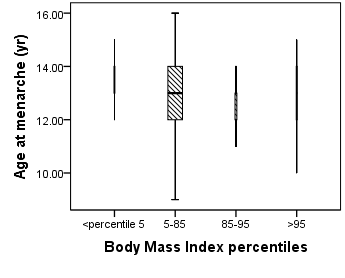


**A. Age at menarche (AM) depending on body mass index (BMI)**

AM had an inverse significant relationship with BMI, as menstruation was occurred earlier in girls with higher BMI.


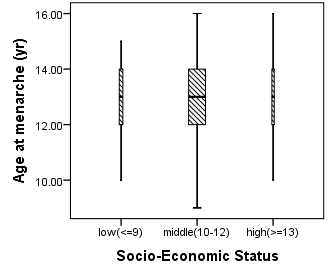


**B. Age at menarche (AM) depending on socio-economic status (SES)**

AM had a statistically significant positive correlation with SES, as with increment in the level of SES, AM increased.
